# The Urine and the Fæces as Vehicles of Typhoid Infection

**Published:** 1899-05-27

**Authors:** 


[1] Lancet, May 20.

### THE URINE AND THE FÆCES AS VEHICLES OF TYPHOID INFECTION.

Dr. Horton-Smith[1] has recently undertaken a very complete investigation as to the respective parts taken by the urine and the fæces in the dissemination of typhoid fever. He concludes that typhoid bacilli occur in the urine of typhoid patients in about 25 per cent. of all cases, and that when they occur they generally remain present for some considerable time, for some weeks or months. They occur first late in the disease, rarely, if ever, before the third week, and may make their first appearance during convalescence. In many of the cases they are present in enormous numbers. In the fæces, on the other hand, typhoid bacilli are present in probably every case; they occur early in the disease, that is, within the first three weeks, and although typhoid stools may contain considerable quantities of them, they rarely, if ever, contain such myriads as may be present in typhoid urine. In regard, then, to the dissemination of the disease, it would appear that the danger arising from the infectious character of the urine, when this is present, is of a much more insidious nature than that depending on infection by the fæces. "The stools, by their colour and odour, at once attract attention, so that the slightest soiling of the linen is at once noticed, and the damage at once rectified, but the same cannot be said of the urine." The moral, as regards the danger run by those engaged in nursing the disease, unless they are as careful about urinary as against fæcal contamination, is sufficiently obvious, and it is also clear that the precautions must not cease with the occurrence of apparent convalescence. The important observation is recorded that in cases in which the presence of typhoid bacilli in the urine has been demonstrated, they have totally disappeared on the administration of urotropin in doses of ten grains three times a day by the mouth.

